# Supplementary material for: Systems genetics analysis of the LXS recombinant inbred mouse strains:Genetic and molecular insights into acute ethanol tolerance
Source: PLoS One. 2020 Oct 23;15(10):e0240253. doi: 10.1371/journal.pone.0240253 (PMC7584226; doi:10.1371/journal.pone.0240253)
Supplement: S1 Fig — Examples of eQTLs that are common or unique as a function of saline or alcohol pretreatment in the LXS RI panel. (PDF) [file pone.0240253.s001.pdf]

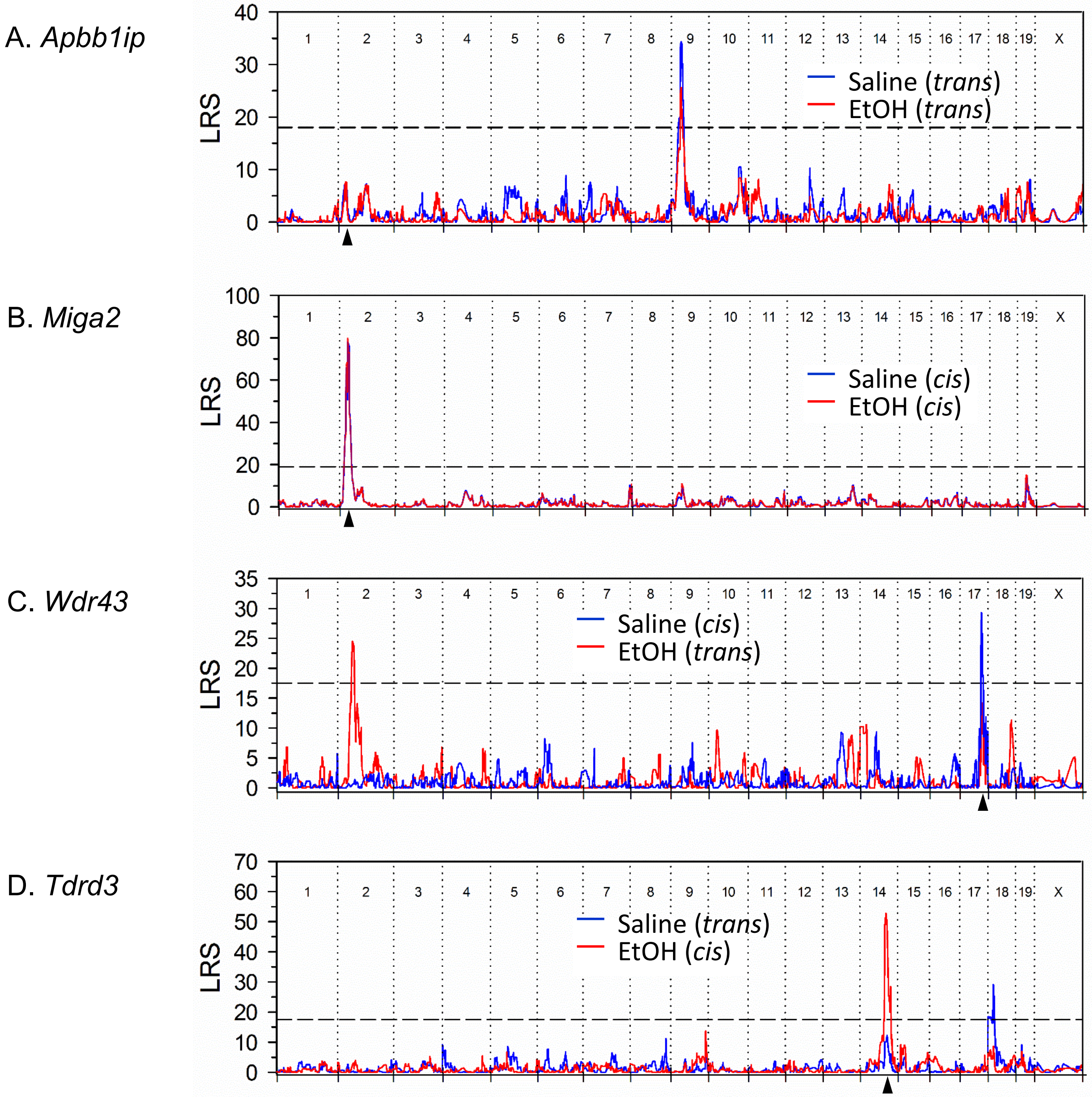

S1 Figure. Examples of eQTLs that are common or unique as a function of saline or alcohol pretreatment in the LXS RI panel.

The dashed horizontal line indicates genome-wide significance threshold as determined by permutation testing ($p<0.05$). Black triangle beneath the X-axis indicates the location of the gene. (A) Common *trans*-regulation. (B) Common *cis*-regulation. (C) and (D) Unique *trans*- and *cis*-regulation.
