# Supplementary material for: Systems genetics analysis of the LXS recombinant inbred mouse strains:Genetic and molecular insights into acute ethanol tolerance
Source: PLoS One. 2020 Oct 23;15(10):e0240253. doi: 10.1371/journal.pone.0240253 (PMC7584226; doi:10.1371/journal.pone.0240253)
Supplement: S2 Fig — REVIGO plot of significantly enriched GO Biological Process terms for genes correlated to Hdac1 (n=446). (PDF) [file pone.0240253.s002.pdf]

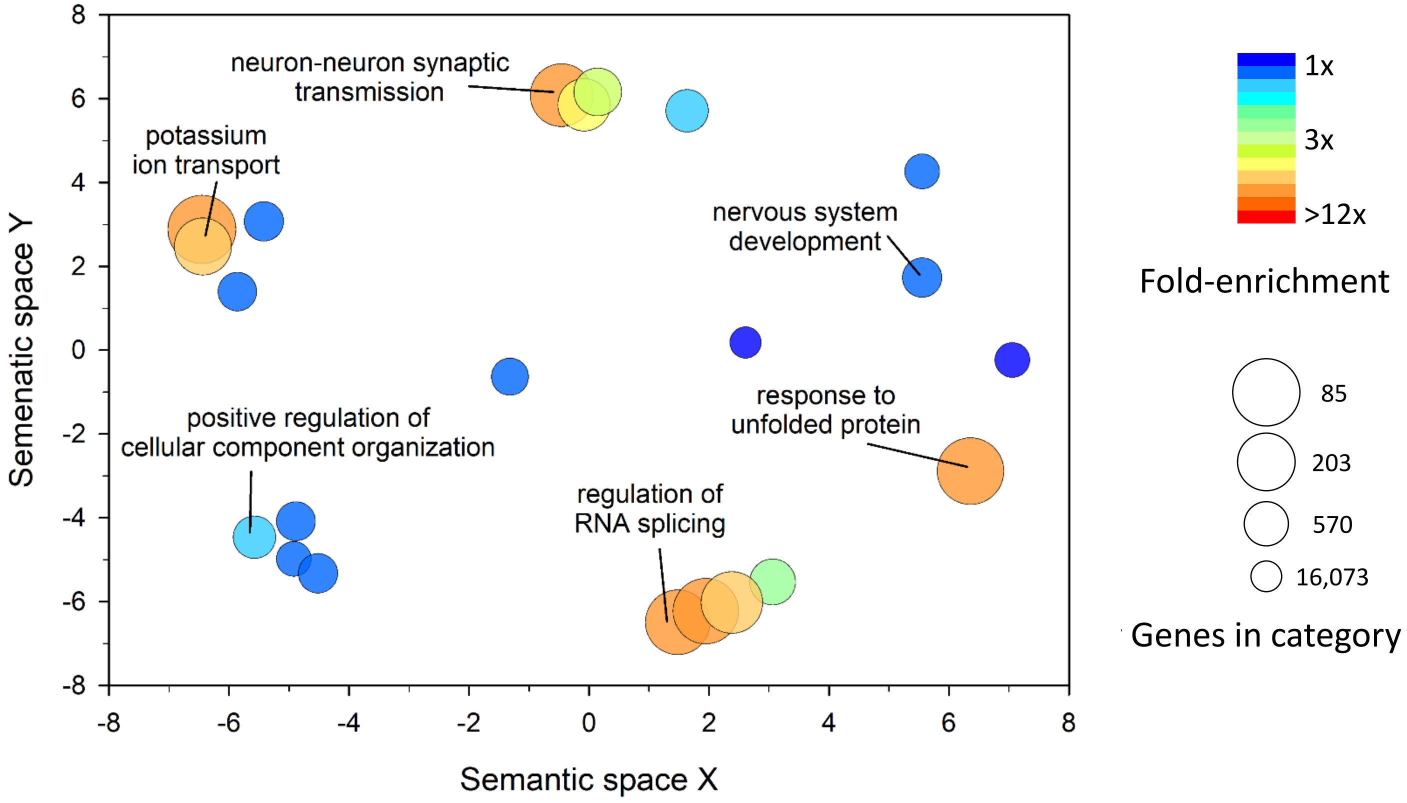

S2 Figure. REVIGO plot of significantly enriched GO Biological Process terms for genes correlated to Hdac1 (n=446).

Fold enrichment is the proportion of term genes found in the input list compared to the proportion of total term genes found in the background. The size of the of the bubble is inversely proportional to the number of genes in the term; *i.e.*, the larger the bubble, the more specific the term.
