# Supplementary material for: Systems genetics analysis of the LXS recombinant inbred mouse strains:Genetic and molecular insights into acute ethanol tolerance
Source: PLoS One. 2020 Oct 23;15(10):e0240253. doi: 10.1371/journal.pone.0240253 (PMC7584226; doi:10.1371/journal.pone.0240253)
Supplement: S1 Table — RNA-seq library and mapping metrics. (PDF) [file pone.0240253.s003.pdf]

S1 Table. RNA-seq library and mapping metrics.

There was a significantly greater number of both total and mapped reads in the alcohol pretreatment group compared to the saline group while the average percent of reads mapped were significantly lower in the alcohol pretreatment group ($p<0.01$). It is unlikely that these results were due to some kind of biological phenomenon and it also is unlikely that it was a batch effect since care was taken to avoid this type of artifact as described in the Methods.

Pretreatment	Average (SEM)	Minimum	Maximum
Saline (40 strains, n=114)			
Total reads (raw)	52.2×10^6 (1.6×10^6) *	22.1×10^6	105.9×10^6
Mapped reads (raw)	35.6×10^6 (1.0×10^6) *	15.7×10^6	67.9×10^6
% Mapped reads	68.9% (0.5%) *	53.0%	78.2%
Ethanol (40 strains, n=118)			
Total reads (raw)	69.2×10^6 (2.0×10^6) *	16.5×10^6	125.7×10^6
Mapped reads (raw)	44.4×10^6 (1.2×10^6) *	11.3×10^6	78.3×10^6
% Mapped reads	64.8% (0.4%) *	50.9%	77.4%

* Saline significantly different from ethanol, $P<0.01$.
