# Supplementary material for: Systems genetics analysis of the LXS recombinant inbred mouse strains:Genetic and molecular insights into acute ethanol tolerance
Source: PLoS One. 2020 Oct 23;15(10):e0240253. doi: 10.1371/journal.pone.0240253 (PMC7584226; doi:10.1371/journal.pone.0240253)
Supplement: S2 Table — Mouse and human transcriptome studies that were compared to the LXS Et-responsive gene list. (PDF) [file pone.0240253.s004.pdf]

S2 Table. Mouse and human transcriptome studies that were compared to the LXS Et-responsive gene list.

Species	Subjects [1]	Treatment [2]	Time after TX	Brain structure(s) [3]	Platform	# Common genes (total from study) [4]	Reference
Mouse	BXD RI	Single acute; 1.8 g/kg, i.p.	4 hrs after injection	Hippocampus	Illumina BeadChips	25 (313)	Baker et al. (2017)
Mouse	WSR	Et vapor, 72 hrs	21 days after last vapor session	PFC	cDNA arrays	6 (86)	Gavin et al. (2018)
Mouse	B6, D2	Single acute; 2 g/kg, i.p.	4 hrs after injection	NAC, PFC, VTA	Affymetrix	25 (253)	Kerns et al. (2005)
Mouse	B6	CIE	Immediate after last vapor session	PFC, NAC, HPC	Affymetrix	39 (458)	Melendez et al. (2012)
Mouse	B6	DID, single 4 hr exposure	Immediate at end of 4 hr exposure	CB, OB, STR, FC, VMB, HIP	cDNA arrays	3 (8)	Mulligan et al. (2011)
Mouse	B6, D2	CIE	72 hrs after last vapor session	CA1, CA3, Prl, ILC, VCX, VTA, NAc, NAs, DMS, CeA, BST	RNA-seq	35 (775)	Mulligan et al. (2017)
Mouse	D2	Multiple acute; 2 g/kg, i.p., 11 days	4 hrs after last injection	Frontal pole, synaptoneurosomes	RNA-seq	226 (4,951)	O'Brien et al. (2018)
Mouse	B6	CH, CI, DID	20-24 hrs after last exposure	PFC	Illumina BeadChips	140 (1,366)	Osterndorff-Kahanek et al. (2013)
Mouse	B6	CIE	0, 8, 120 hrs after last vapor session	AMY, PFC, NAC	Illumina BeadChips	205 (2,970)	Osterndorff-Kahanek et al. (2015)
Mouse	B6	CIE	0, 8, 72 hrs after last vapor session	PFC, NAC, HPC, BNST, CEA	Affymetrix	434 (5,819)	Smith et al. (2016)
Mouse	BXD RI	CIE	72 hrs after last vapor session	PFC, NAC	Affymetrix	182 (2,314)	van der Vaart et al. (2017)
Mouse	B6, D2, BXD RI	Single acute Et; 1.8 g/kg, i.p.	4 hrs after injection	PFC, VMB, NAc	Affymetrix	224 (2,749)	Wolen et al. (2012)
Human	DSM-IV	Chronic drinking	Postmortem	Prefrontal cortex	Affymetrix	5 (53)	Iwamoto et al. (2004)

Species	Subjects [1]	Treatment [2]	Time after TX	Brain structure(s) [3]	Platform	# Common genes (total from study) [4]	Reference
Human	NHMRC/ WHO	Chronic drinking	Postmortem	Superior frontal cortex	cDNA arrays, Affymetrix	7 (84)	Lewohl et al. (2000)
Human	NHMRC	Chronic drinking	Postmortem	Superior frontal cortex	cDNA arrays	21 (325)	Liu et al. (2006)
Human	NHMRC	Chronic drinking	Postmortem	Frontal cortex	cDNA arrays	69 (1,124)	Liu et al. (2007)
Human	NHMRC/ WHO	Chronic drinking	Postmortem	Frontal cortex, motor cortex	cDNA arrays	5 (35)	Mayfield et al. (2002)
Human	Alcoholic	Chronic drinking	Postmortem	Hippocampus	Affymetrix	59 (685)	McClintick et al. (2013)
Human	DSM-IV	Chronic drinking	Postmortem	CTX, BLA, CNA	Illumina BeadChips	362 (4,755)	Ponomarev et al. (2012)
Human	DSM-IV	Chronic drinking	Postmortem	Temporal cortex	Affymetrix	5 (148)	Sokolov et al. (2003)
Human	DSM-IV	Chronic drinking	Postmortem	Hippocampus	RNA-seq	17 (335)	Zhou et al. (2011)

[1] Mouse: B6, C57BL/6; D2, DBA/2; WSR, Withdrawal Seizure Resistant; RI, Recombinant Inbred strains.
Human criteria for alcoholism: NHMRC, National Health and Medical Research Council; WHO, World Health Organization; DSM-IV, Diagnostic and Statistical Manual of Mental Disorders, Fourth Edition; Alcoholic, no specific criteria mentioned.

[2] Mouse: Et vapor, CH, CI, and CIE all designate that the mice spent some portion of time (days to weeks) in an ethanol vapor chamber; DID, drinking in the dark. Specific procedures varied by study.
Human: each study included a table of demographics and other information about the subjects, including drinking history.

[3] Abbreviations and terminology as used by the authors:
STR, striatum; DMS, dorsomedial striatum; NAC, nucleus accumbens; NAs, nucleus accumbens shell; NAc, nucleus accumbens core.
AMY, amygdala; BLA, basolateral nucleus of amygdala; CAN/CeA/CEA, central nucleus of amygdala.
PFC, prefrontal cortex; CTX, superior frontal cortex; Prl, prelimbic cortex; ILC, infralimbic cortex; VCX, primary visual cortex.
VMB, ventral midbrain; VTA, ventral tegmental area.
HPC, hippocampus; CA1/CA3,hippocampal structures.
BST/BNST, bed nucleus stria terminalis.
CB, cerebellum.
OB, olfactory bulbs.

[4] Total from study (in parentheses) indicates the total number of genes that were identified as significantly differentially expressed due to ethanol treatment based on the authors' criteria.

References

Baker JA, Li J, Zhou D, Yang M, Cook MN, Jones BC, Mulligan MK, Hamre KM, Lu L (2017) Analyses of differentially expressed genes after exposure to acute stress, acute ethanol, or a combination of both in mice. Alcohol 58:139-151.

Gavin DP, Hashimoto JG, Lazar NH, Carbone L, Crabbe JC, Guizzetti M (2018) Stable Histone Methylation Changes at Proteoglycan Network Genes Following Ethanol Exposure. Frontiers in Genetics 9.

Iwamoto K, Bundo M, Yamamoto M, Ozawa H, Saito T, Kato T (2004) Decreased expression of NEFH and PCP4/PEP19 in the prefrontal cortex of alcoholics. Neuroscience Research 49:379-385.

Kerns RT, Ravindranathan A, Hassan S, Cage MP, York T, Sikela JM, Williams RW, Miles MF (2005) Ethanol-responsive brain region expression networks: implications for behavioral responses to acute ethanol in DBA/2J versus C57BL/6J mice. The Journal of neuroscience : the official journal of the Society for Neuroscience 25:2255-2266.

Lewohl JM, Wang L, Miles MF, Zhang L, Dodd PR, Harris RA (2000) Gene Expression in Human Alcoholism: Microarray Analysis of Frontal Cortex. Alcoholism: Clinical and Experimental Research 24:1873-1882.

Liu J, Lewohl JM, Harris RA, Dodd PR, Mayfield RD (2007) Altered Gene Expression Profiles in the Frontal Cortex of Cirrhotic Alcoholics. Alcoholism: Clinical and Experimental Research 31:1460-1466.

Liu J, Lewohl JM, Harris RA, Iyer VR, Dodd PR, Randall PK, Mayfield RD (2006) Patterns of gene expression in the frontal cortex discriminate alcoholic from nonalcoholic individuals. Neuropsychopharmacology 31:1574-1582.

Mayfield RD, Lewohl JM, Dodd PR, Herlihy A, Liu J, Harris RA (2002) Patterns of gene expression are altered in the frontal and motor cortices of human alcoholics. Journal of neurochemistry 81:802-813.

McClintick JN, Xuei X, Tischfield JA, Goate A, Foroud T, Wetherill L, Ehringer MA, Edenberg HJ (2013) Stress-response pathways are altered in the hippocampus of chronic alcoholics. Alcohol 47:505-515.

Melendez RI, McGinty JF, Kalivas PW, Becker HC (2012) Brain region-specific gene expression changes after chronic intermittent ethanol exposure and early withdrawal in C57BL/6J mice. Addict Biol 17:351-364.

Mulligan MK, Mozhui K, Pandey AK, Smith ML, Gong S, Ingels J, Miles MF, Lopez MF, Lu L, Williams RW (2017) Genetic divergence in the transcriptional engram of chronic alcohol abuse: A laser-capture RNA-seq study of the mouse mesocorticolimbic system. Alcohol 58:61-72.

Mulligan MK, Rhodes JS, Crabbe JC, Mayfield RD, Harris RA, Ponomarev I (2011) Molecular profiles of drinking alcohol to intoxication in C57BL/6J mice. Alcohol Clin Exp Res 35:659-670.

O'Brien MA, Weston RM, Sheth NU, Bradley S, Bigbee J, Pandey A, Williams RW, Wolstenholme JT, Miles MF (2018) Ethanol-Induced Behavioral Sensitization Alters the Synaptic Transcriptome and Exon Utilization in DBA/2J Mice. Frontiers in Genetics 9.

Osterndorff-Kahanek E, Ponomarev I, Blednov YA, Harris RA (2013) Gene expression in brain and liver produced by three different regimens of alcohol consumption in mice: comparison with immune activation. PLoS ONE 8**:**e59870.

Osterndorff-Kahanek EA, Becker HC, Lopez MF, Farris SP, Tiwari GR, Nunez YO, Harris RA, Mayfield RD (2015) Chronic ethanol exposure produces time- and brain region-dependent changes in gene coexpression networks. PLoS One 10**:**e0121522.

Ponomarev I, Wang S, Zhang L, Harris RA, Mayfield RD (2012) Gene coexpression networks in human brain identify epigenetic modifications in alcohol dependence. The Journal of neuroscience : the official journal of the Society for Neuroscience 32**:**1884-1897.

Smith ML, Lopez MF, Archer KJ, Wolen AR, Becker HC, Miles MF (2016) Time-Course Analysis of Brain Regional Expression Network Responses to Chronic Intermittent Ethanol and Withdrawal: Implications for Mechanisms Underlying Excessive Ethanol Consumption. PLoS One 11**:**e0146257.

Sokolov BP, Jiang L, Trivedi NS, Aston C (2003) Transcription profiling reveals mitochondrial, ubiquitin and signaling systems abnormalities in postmortem brains from subjects with a history of alcohol abuse or dependence. Journal of Neuroscience Research 72**:**756-767.

van der Vaart AD, Wolstenholme JT, Smith ML, Harris GM, Lopez MF, Wolen AR, Becker HC, Williams RW, Miles MF (2017) The allostatic impact of chronic ethanol on gene expression: A genetic analysis of chronic intermittent ethanol treatment in the BXD cohort. Alcohol 58**:**93-106.

Wolen AR, Phillips CA, Langston MA, Putman AH, Vorster PJ, Bruce NA, York TP, Williams RW, Miles MF (2012) Genetic dissection of acute ethanol responsive gene networks in prefrontal cortex: functional and mechanistic implications. PLoS ONE 7**:**e33575.

Zhou Z, Yuan Q, Mash DC, Goldman D (2011) Substance-specific and shared transcription and epigenetic changes in the human hippocampus chronically exposed to cocaine and alcohol. Proceedings of the National Academy of Sciences 108**:**6626-6631.
